# Treatment of thoraco-lumbar curves in adolescent females affected by idiopathic scoliosis with a progressive action short brace (PASB): assessment of results according to the SRS committee on bracing and nonoperative management standardization criteria

**DOI:** 10.1186/1748-7161-4-21

**Published:** 2009-09-18

**Authors:** Angelo G Aulisa, Vincenzo Guzzanti, Marco Galli, Carlo Perisano, Francesco Falciglia, Lorenzo Aulisa

**Affiliations:** 1 Orthopaedic Department, Children's Hospital Bambino Gesù, Institute of Scientific Research, P.zza S. Onofrio, 4 - 00165 Rome, Italy; 2 University of Cassino, Strada Folcara, 4 - 03043 Cassino (FR), Italy; 3 Orthopaedic Department, Children's Hospital Bambino Gesù, Institute of Scientific Research, P.zza S. Onofrio, 4 - 00165 Rome, Italy; 4 Department of Orthopaedics, "A. Gemelli" Hospital, Università Cattolica del Sacro Cuore, Largo A. Gemelli, 1 - 00168, Rome, Italy

## Abstract

**Background:**

The effectiveness of conservative treatment of scoliosis is controversial. Some studies suggest that brace is effective in stopping curve progression, whilst others did not report such an effect.

The purpose of the present study was to effectiveness of Progressive Action Short Brace (PASB) in the correction of thoraco-lumbar curves, in agreement with the Scoliosis Research Society (SRS) Committee on Bracing and Nonoperative Management Standardisation Criteria.

**Methods:**

Fifty adolescent females (mean age 11.8 ± 0.5 years) with thoraco-lumbar curve and a pre-treatment Risser score ranging from 0 to 2 have been enrolled. The minimum duration of follow-up was 24 months (mean: 55.4 ± 44.5 months). Antero-posterior radiographs were used to estimate the curve magnitude (C_M_) and the torsion of the apical vertebra (T_A_) at 5 time points: beginning of treatment (t_1_), one year after the beginning of treatment (t_2_), intermediate time between t_1 _and t_4 _(t_3_), end of weaning (t_4_), 2-year minimum follow-up from t_4 _(t_5_). Three situations were distinguished: curve correction, curve stabilisation and curve progression.

The Kruskal Wallis and Spearman Rank Correlation tests have been used as statistical tests.

**Results:**

C_M _mean value was 29,30 ± 5,16 SD at t_1 _and 14,67 ± 7,65 SD at t_5_. T_A _was 12.70 ± 6,14 SD at t_1 _and 8,95 ± 5,82 at t_5_. The variation between measures of Cobb and Perdriolle degrees at t_1,2,3,4,5 _and between C_M _t_5_-t_1 _and T_A _t_5_-t_1 _were significantly different.

Curve correction was accomplished in 94% of patients, whereas a curve stabilisation was obtained in 6% of patients.

**Conclusion:**

The PASB, due to its peculiar biomechanical action on vertebral modelling, is highly effective in correcting thoraco-lumbar curves.

## Background

The treatment of adolescent idiopathic scoliosis (AIS) is aimed at stopping the progression of the deformity, and improving the aesthetic appearance, trunk balance and quality of life [1].

However, the effectiveness of the various therapeutic approaches is still controversial, mainly because of the incomplete knowledge of the aetiology and pathogenesis of the deformity [2]. Furthermore, until recently, the lack of uniform criteria for the inclusion of participants and evaluation of results made the comparison of the studies difficult to compare. Some studies have suggested that bracing may represent an effective strategy to treat AIS, as it can stop scoliosis progression and reduce the need for surgery [3-11]. However, other studies have not confirmed such effects [12-16]. In addition, long-term correction of idiopathic scoliosis has not been achieved with most braces in use.

Since 1976, we have been treating thoraco-lumbar and lumbar idiopathic curves with the Progressive Action Short Brace (PASB), a custom-made thoraco-lumbar-sacral orthosis (TLSO) brace of original design, devised by Dr. Lorenzo Aulisa. The PASB being a low brace is indicated only for the treatment of thoraco-lumbar and lumbar curves.

**Figure 1:**
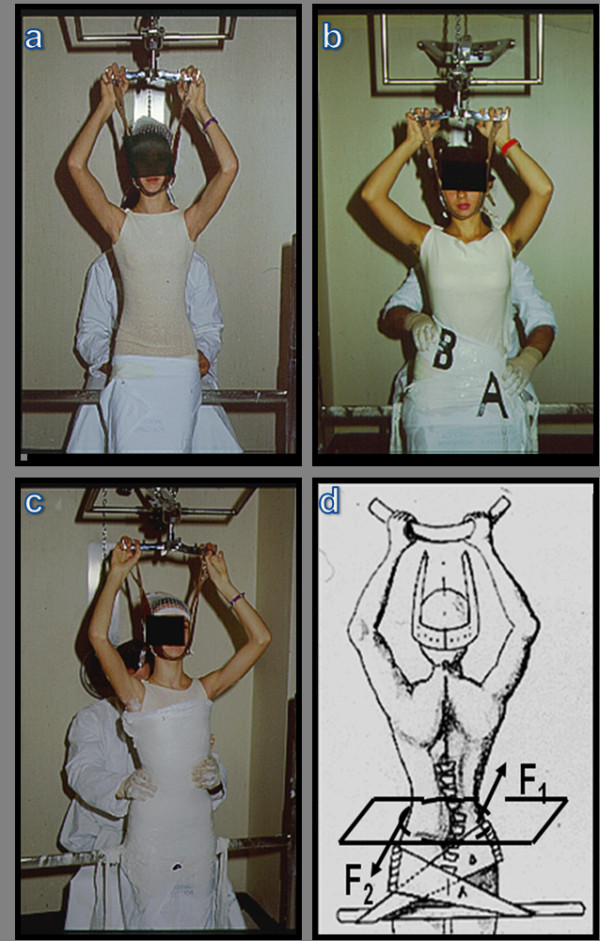
**Brace or plaster cast custom made**. a. The patient, in light traction, is positioned with hip and knees lightly bent, in order to obtain the correction of the hyperlordosis. b. Lateral deflection is achieved by applying a plaster strap just beneath the apical vertebra (B). A second strap stabilises the pelvis (A). c. After the plaster cast is complited, the operator appliesa twisting moment. d. The direction of the rotation produced by the couple of forces is opposite to the direction of the vertebral torsion of the scoliotic curve. This allows to obtain transverse sections represented by asymmetrical ellipses.

**Figure 2:**
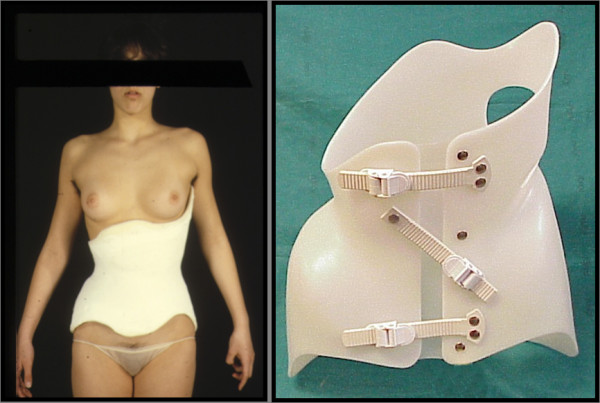
**The finishing touches of the plaster cast establish the same geometry of the plastic brace**.

The brace is informed by the principle that a constrained spine dynamics can achieve correction of a curve, by inverting the abnormal load distribution during growth [17-20]. The practical application of the biomechanical principles of the PASB is achieved through two operative phases. A plaster cast phase precedes the brace application. At this stage, external forces are imparted to correct the flexible component of the deformity. Elongation is exerted by suspending the patient with a head-halter while sitting on a horizontal bar. Lateral deflection is achieved by applying a plaster strap just beneath the apical vertebra. A second strap stabilises the pelvis. More straps are laid down in the same fashion. Finally, a derotating force couple is imparted, which is clockwise or anticlockwise-oriented according to whether the curve is right convex or left convex, respectively. This procedure allows to obtain transversal sections represented by asymmetric ellipsis (figure 1). The finishing touch of the cast establishes the real geometry of the plastic brace (figure 2). One or sometimes two casts, in relation to the curve rigidity, are manufactured before switching to the custom-made polypropylene orthosis.

In the second phase, a plaster mould is used for the custom made PASB manufacturing. The brace mode of action depends on its peculiar geometry, that is determined by the outlines of the free ends and by a redistribution of the volumes.

**Figure 3:**
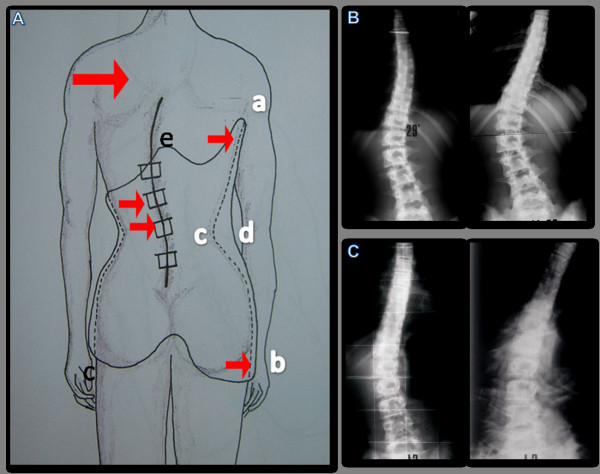
**Dynamics bound by the geometry of the brace**. From the concave side, the brace extends from the throcanteric region (A) upto the superior neutral vertebra (b. Therefore, the flection toward the deformity is opposed. The presence of a free space (c-d) between the iliac crest and the upper vertebral limit favours the spine realignment along the cefalo-caudal axis plane (A). The shape of the concave side without (b) and with the brace (c) shows the remarkable diversity between the dynamics of the free and of bound spine.

In the coronal plane the upper margin of the brace on the concave side prevents homolateral bending. The opposite superior margin ends just beneath the apical vertebra (figure 3).

**Figure 4:**
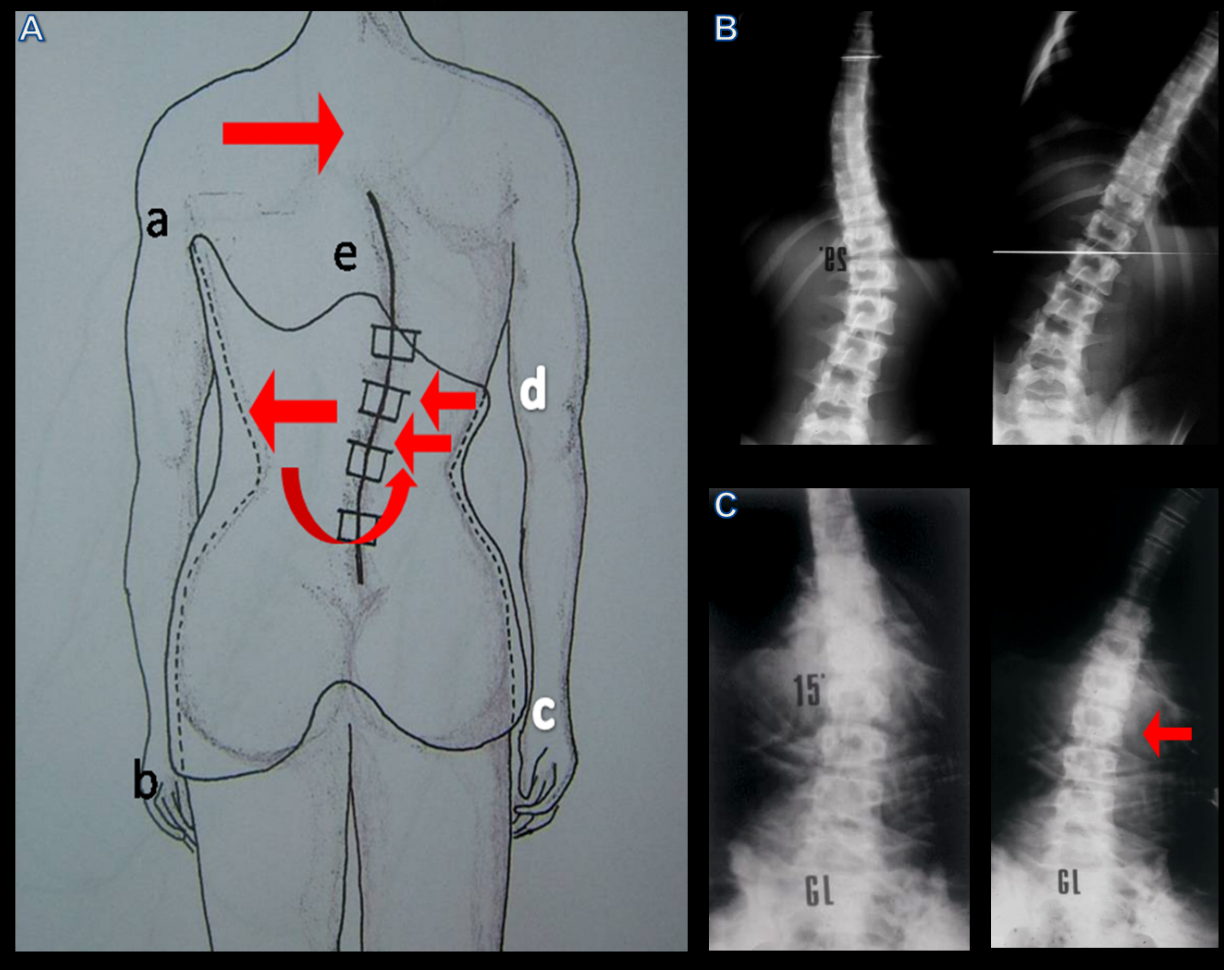
**Dynamics bound by the geometry of the brace**. On the convex side, the superior margin ends under the apex vertebra, allowing the lateral flection movement to induce the deflection of the curve and the realignment of the spine under the apex vertebra. The posterior edge is modelled with a central active prominence which is uncomfortable and compels the patient to perform an anterior translation that allows the hyperlordosis correction (A). Such a profile imposes a particular dynamics, for which the anterior flection movements can be performed only together with a lateral flection and a rotation in the direction of the correction of the curve. The bending X-Ray exam executed on the convexity side without (b) and with the brace (c) confirms the theoretical presuppositions showing the remarkable behaviour diversity between the dynamics of the free and the bound spine.

It should be considered that deflection of the inferior tract of a curved elastic structure, which is fixed at either end, causes straightening of its upper tract. Therefore, whenever the patient bends to the convex side, the spine is deflected (figure 4).

In the sagittal plane, the inferior margins reach the pelvitrochanteric region, and thus stabilises the pelvis.

**Figure 5:**
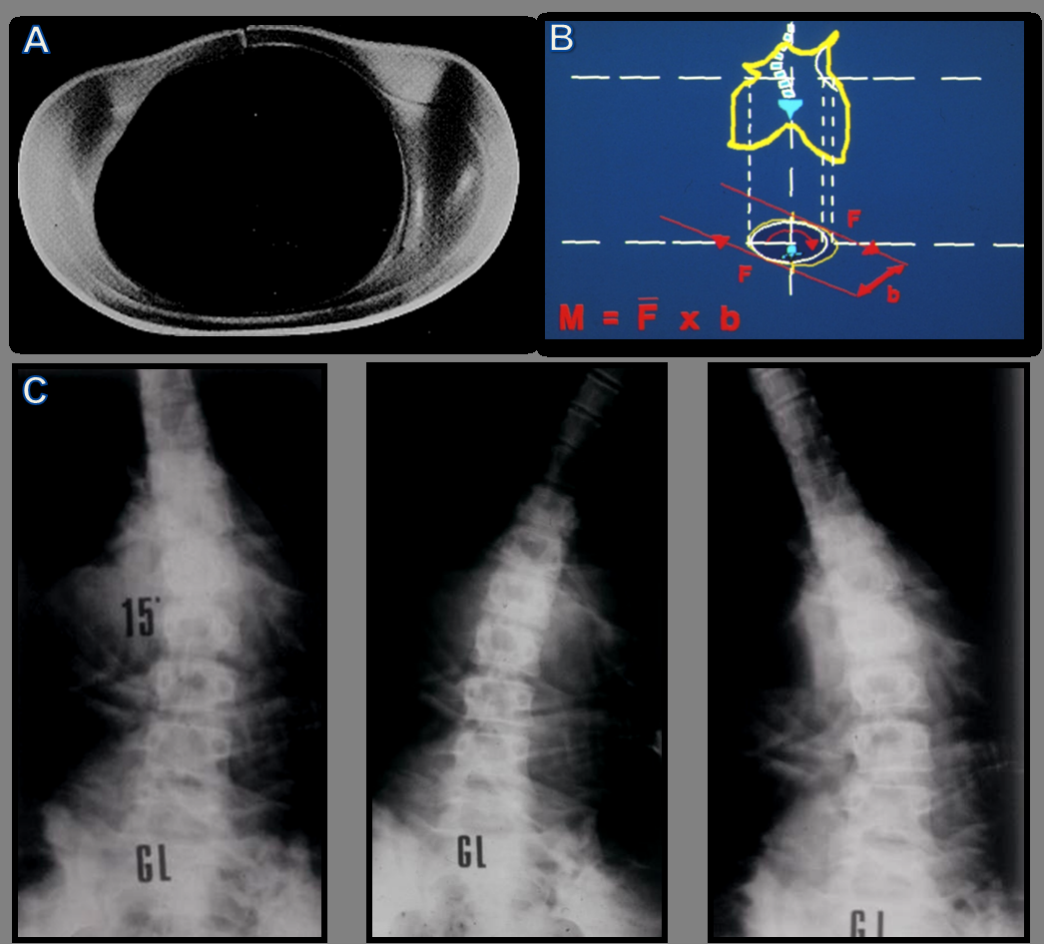
**Dynamics bound by the geometry of the brace**. The trasverse section of the brace (A), elliptical and asymmetrical up to the plan of the pelvic Hold, produces twisting moments opposed to the direction of the rotation of the vertebrae included in the curve (b). The bending X-ray (c) shows the efficacy of the twisting action produced by the brace for all movements of the trunk.

The transverse section above the pelvic grip are asymmetrical ellipses, allowing the spine to rotate towards to concave side only, such that derotating moments are constantly generated (figure 5). In the sagittal plane the brace is contoured so as to reduce the lumbar lordosis.

The PASB, by permitting only those movements hindering the progression of the curve, is able to bring about corrective forces that are not bound to be dissipated.

Early clinical results showed that a stable correction of the curves could be obtained. These results were confirmed in a prospective study performed in patients treated with the PASB [21,22].

In the present study, we have reviewed our previous series, in order to fulfil the recent international Standardization Criteria put forward by the SRS Committee for Bracing and Non-operative Management [23], following the guidelines on standard management of idiopathic scoliosis with corrective braces in everyday clinics and in clinical research[24].

## Methods

Fifty adolescent females out of 77 patients fulfilled the inclusion criteria of the SRS Committee. Twenty-seven patients were excluded because the brace had been prescribed at 8-9 years of age (n = 8), the magnitude the primary curve was not comprised between 25 and 40° Cobb (n = 7), the menarche had occorred over 1 year before (n = 2), and because of a lack of compliance (n = 10). Management of patients was performed according to the guide lines of the SOSORT [24].

All patients presented with a single major thoraco-lumbar curve, whose magnitude was comprised between 25° and 40° Cobb. The age at the beginning of treatment ranged between 10 and 12 years, with a Risser score from 0 to 2. Full-time (i.e., 22 hours per day) bracing was instituted for all progressive curves. Compliance to the treatment was established via in-person interviews with both the patients and their relatives.

Curve progression was assessed on two consecutive X-rays taken at 6 months interval. Progression was defined as an increase greater than 5° in both curve magnitude (Cobb's method) and apical torsion (Perdriolle's method) [25,26].

Weaning was started when ring-apophyses fusion was seen to begin on a latero-lateral view X-ray and consisted of 2-4 hours bracing reduction at 4-month intervals. The curve response to progressive part-time bracing was observed on an antero-posterior view standing radiograph after the patient had been out of the brace for 5 hours. Out-of-brace hours were not reduced or even increased if the curve was not stable. Treatment was ended when ring-apophyses fusion was complete on a latero-lateral X-ray [27]. A minimum follow-up of 2 years after the end of treatment was performed.

Patients not bracing for the planned hours have been excluded from the analysis. The daily hours of bracing (i.e. Max 22, min 18) have been defined for each patient and their parents both in relation to the subject clinical needs and availability. In order to maximize the compliance, patients have been always followed by the same doctor. Furthermore, checks have been performed every 2 months till Risser 3 and later on every 3 months. Frequent checks allow to verify and to implement compliance establishing an open and friendly relationship with the patients. Close checks are also necessary to maximise bracing effectiveness over the time.

X-ray evaluation was performed at conventional times (t): beginning of treatment (t_1_), one year after the beginning of treatment (t_2_), intermediate time between t_1 _and t_4 _(t_3_), end of weaning (t_4_), 2-year minimum follow-up from t_4 _(t_5_), in t_2_-t_3_-t_4 _the X-rays were carried out with the brace, because in this way we can evaluate the effectiveness of the brace. For each patient an A.P (anterior posterior) and LL (latero-lateral) view standing X-rays of the whole spine were available. All radiographs were taken at our Institute at a 2-meter distance on a 36 × 91 cm film. The AP view was used to determine the patient's skeletal age (Risser's sign) and to obtain to the curve magnitude (C_M_, Cobb's method) and torsion of the apical vertebra (T_A_: Perdriolle's method). Measurements were independently obtained by two observers. The end-vertebrae were pre-selected to reduce inter-observer error [25]. C_M_, T_A _variations at t_1_, t_2_, t_3_, t_4 _and t_5 _were assessed by the Kruskal-Wallis H test. Correlations between changes of C_M _at t_5 _and t_1 _(C_M _t_5_-t_1_), and T_A _at t_5 _and t_1 _(T_A _t5-tl), as well as patient's age at t_1 _were assessed via the Spearman's rank correlation test. The same test was performed to determine correlations between C_M _t_5_-t_1_, T_A _t_5_-t_1 _and Risser's sign at t_1_. Finally, results were analysed in relation to C_M _t_5_-t_1 _at follow-up, assuming that C_M _t_5_-t_1 _had not to be within the Cobb's method ± 5 range error [25]. Three situations were distinguished: curve correction (C_M _t_5_-t_1 _≤ -5° Cobb), curve stabilisation (C_M _t_5_-t_1 _≥ -5 and ≤ 5° Cobb) and curve progression (C_M _t_5_-t_1 _> 5° Cobb). Significance was set at p < 0.05. Results are presented as mean ± standard deviation (SD).

## Results

**Figure 6:**
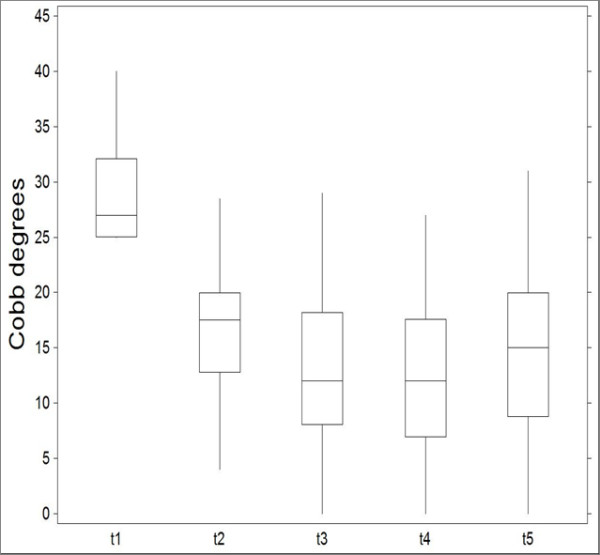
**Variation of Cobb degrees at t_1,2,3,4,5_**.

**Figure 7:**
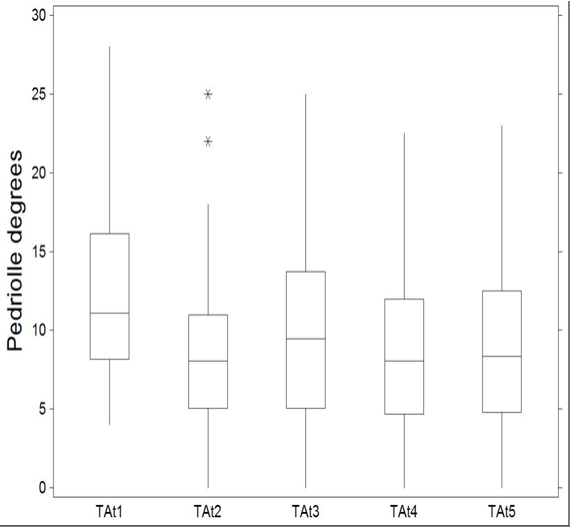
**Variation of Perdriolle degrees at t_1,2,3,4,5_**.

The mean age was 11.8 ± 0.5 years and 18.6 ± 1.3 years at t_1 _and t_5_, respectively. The mean duration of treatment was 58.8 ± 21.9 months, whereas the mean duration of the follow-up was 55.4 ± 44.5 months (range 24 - 204 months). C_M _mean value was 29.30 ± 5.16 at t_1 _(range 25-40) and 14.67 ± 7.56 t t_5_. T_A _was 12.70 ± 6.14 at t_1 _and 8.95 ± 5.82 at t_5 _(figure 6 and 7).

Measures of Cobb degrees at were significantly different across t_1,_t_2,_t_3,_t_4,_t_5 _(KW = 111.902; P < 0.0001, table 1). Significant differences were also detected when comparing measures of Pedriolle degrees across t_1,_t_2,_t_3,_t_4,_t_5 _(KW = 16.9880; P < 0.01, table 2).

**Table 1:** Kruskal-Wallis All-Pairwise Comparisons Test (Alpha 0.05, Critical Z Value 2,807, Critical Value for Comparison 40.597)

**Variable**	**Mean**	**C**_**M **_**t**_**1**_	**C**_**M **_**t**_**2**_	**C**_**M **_t_**3**_	**C**_**M **_t_**4**_
**C_M _t_1_**	218.33				

**C_**M **_t_**2**_**	125.18	93.15*			

**C_**M **_t_**3**_**	91.280	127.05*	33.90		

**C_**M **_t_**4**_**	86.870	131.46*	38.31	4.41	

**C_**M **_t_**5**_**	105.84	112.49*	19.34	14.56	18.97

**Table 2:** Kruskal-Wallis All-Pairwise Comparisons Test (Alpha 0.05, Critical Z Value 2,807, Critical Value for Comparison 40.597)

**Variable**	**Mean**	**T_**A **_t_**1**_**	**T_**A **_t_**2**_**	**T_**A **_t_**3**_**	**T_**A **_t_**4**_**
**T_**A **_t_**1**_**	161.00				

**T_**A **_t_**2**_**	110.41	50.59*			

**T_**A **_t_**3**_**	127.33	33.67	16.92		

**T_**A **_t_**4**_**	110.09	50.91*	0.32	17.24	

**T_**A **_t_**5**_**	118.67	42.33*	8.26	8.66	8.58

The following correlations were calculated: C_M _t_5_-t_1 _and T_A _t_5_-t_1 _(r_s _= 0.32, p < 0.025); C_M _t_5_-t_1 _and patient's age at t_1 _(r_s _= 0.01, ns); C_M _t_5_-t_1 _and Risser'sign at t_1 _(r_s _= 0.07, ns); T_A _t_5_-t_1 _and patient's age at t_1 _(r_s _= -0.11, ns); T_A _t_5_-t_1 _and Risser's sign at t_1 _(r_s _= 0.19, ns). Forty-seven out of the 50 patients (94%) obtained a curve correction (mean C_M _t_5_-t_1 _was -13.97 ± 5,25° Cobb), whilst a curve stabilisation was achieved in 3 patients (6%) (mean C_M _t_5_-t_1 _was -4,67 ± 0.58° Cobb). None of the patients presented a curve progression (>5°) after brace treatment.

## Discussion and Conclusion

Recent studies comparing the natural history of idiopathic scoliosis with the results of bracing have suggested that the orthotic treatment can modify the natural history of mild scoliosis [3,4]. Furthermore, a metanalysis indicates that bracing is an effective non-surgical tool for treating scoliosis [5]. Despite a reappraisal of bracing as a rational and effective treatment, no substantial advances to improve the biomechanical performance of braces have been achieved. Particularly, two main problems remain unsolved. Firstly, most braces in use are capable of exerting a bending moment but not an effective torque [28]. Since several studies have pointed out that rotation is the main pathomechanical factor responsible for the progression of scoliosis [29,30], the lack of a derotating moment may negatively affect the mechanical effectiveness of braces. The second problem is that orthotics impart external forces, which undergo dissipation when they are applied to a viscoelastic structure such as the spine, owing to the creep and relaxation phenomena. This ensues that, for orthoses to be more effective, a constant reiteration of forces should be pursued, which is unpractical for cost-effective and for compliance reasons. The PASB sets itself as an original orthosis for the treatment of thoracolumbar and lumbar idiopathic curves. The PASB biomechanics is in fact based on the principle that the spine dynamics can be aptly constrained in each of the three space planes. This ensures that only the degrees of freedom whose direction is opposite to that producing an aggravation of the deformity are permitted [18]. Such an approach allows to achieve the correction and not only stabilisation of a curve, by inverting the abnormal load distribution throughout the growth [21]. The results obtained in the present study, reviewed according the international standardization criteria, would further validate the biomechanical premises underlying the PASB action. Mean C_M _t_5_-t_1 _was -13.97 ± 5,25° Cobb in 47 (94%) out of the 50 patients, indicating that a curve correction was obtained. In the remaining 3 patients, a curve stabilisation was achieved (6%), the mean C_M _t_5_-t_1 _being -4,67 ± 0.58° Cobb.

Importantly, none of the patients presented a curve progression ≥6° Cobb after treatment and skeletal maturity, thus obviating the need for surgery treatment in our case series.

**Figure 8:**
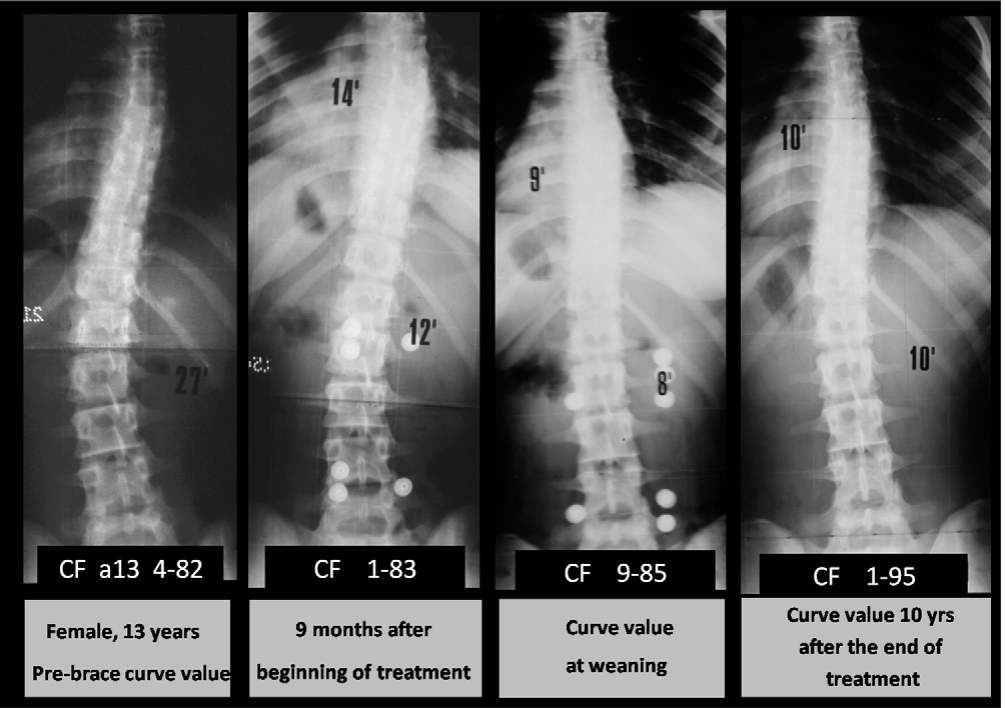
**Typical trend of scoliosis in treattment with PASB**.

**Figure 9:**
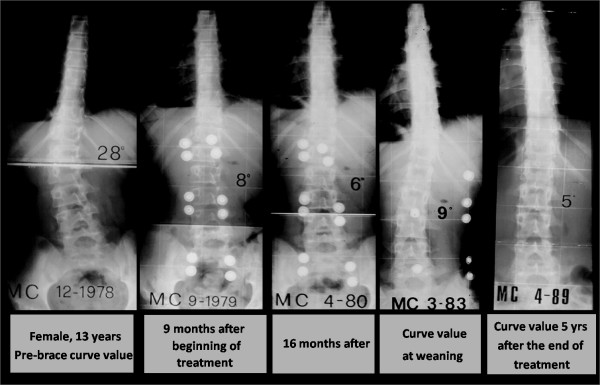
**Example of the effectiveness of the derotation actions with inversion of prossimal vertebrae rotation and curve doubling**.

In addition, our results indicate that the P.A.S.B. not only reduces the curve angular value in the coronal plane, but is also able to derotate the vertebrae included in the curve. Both these factors may be regarded as instrumental in attaining a stable curve correction. In fact, in our series a significant correlation was detected between C_M _t_5_-t_1 _- T_A _t_5_-t_1 _(r_s _= 0.32, p < 0.025), demonstrating the effectiveness of the treatment on both parameters. In contrast, we did not detect a correlation between the age of the patient (either anagraphical or biological, as determined via the Risser score) at the beginning of the treatment and the degree of correction achieved. Maintenance of angular reduction in the course of growth is essential if a stable correction of the deformity is to be achieved. This entails that the brace has to exert forces capable of inverting the asymmetrical growth of the scoliotic spin, such to direct the vertebral growth according to as an eumorphic model as possible. In fact, it has been shown that the vertebral growth occurs according to the Huter-Volkman's law [31]. In addition, in a previous study, we proposed a theoretical model suggesting that the disc elastic behaviour to torsion, that is its aptitude to load transrnission, varies according to other factors, including its location and its strain status, in relation to the subject's age [17]. As far as vertebral rotation is concerned, the application of a derotating torque throughout the growing years would consequently affect the spine modelling in the transverse plane as well (figure 8). Therefore, our study indicates that the PASB, due to its peculiar biomechanical action on vertebral modelling, may achieve a stable correction of thoracolumbar curves in a high percentage of patients affected by idiopathic scoliosis (figure 9).

We compared our results with those reported by Lonstein and Carlson [3], who analyzed the natural history of AIS in non-treated adolescent males and females. The authors showed that those subjects with curves ranging between 20 and 29° Cobb and a Risser score of 0-1, displayed a progression rate of 68%. Strikingly, in our series, among the 18 cases with Risser 0-1 and curves of 20-29° Cobb, no progression was observed in 16 cases (88,9%), with a correction >5° Cobb. The remaining 2 cases (11,1%) achieved a curve stabilisation, with final Cobb's degrees ≤5.

Moreover, we compared our results with those obtained by Nachemson and Peterson [4]. The authors studied girls affected by idiopathic scoliosis with curves between 25° and 35° Cobb and with curve apex between T8 and L1, who had worn a underarm thoraco-lumbar sacral orthosis (TLSO). Their results indicate that a curve progression did not occur in 74% of the patients wearing the orthosis. In our series, 29 patients had curves between 25° and 35° Cobb, with a curve apex between T8 and L1. Correction was achieved in 27 cases (93,1%), whereas 2 cases (6,9%) displayed a stabilisation. Although, our sample was smaller than that analysed by Nachemson and Peterson, participants in the two studies were similar, except for the fact that subjects with Risser 3 or 4 were excluded in our study. In conclusion, results obtained by using the PASB are better than those achieved in the other two studies.

## Competing interests

The authors declare that they have no competing interests.

## Authors' contributions

All authors contributed equally to this work, all authors read and approved the final manuscript.
